# JAK/STAT in leukemia: a clinical update

**DOI:** 10.1186/s12943-023-01929-1

**Published:** 2024-01-26

**Authors:** Dong Liang, Qiaoli Wang, Wenbiao Zhang, Hailin Tang, Cailu Song, Zhimin Yan, Yang Liang, Hua Wang

**Affiliations:** 1 State Key Laboratory of Oncology in South China, Guangdong Provincial Clinical Research Center for Cancer, Sun Yat-Sen University Cancer Center, Guangzhou, China; 2 Department of Hematology, The First Affiliated Hospital of Gannan Medical University, Ganzhou, China

**Keywords:** JAK/STAT signaling pathway, Leukemia, JAK/STAT inhibitors

## Abstract

Over the past three decades, considerable efforts have been expended on understanding the Janus kinase/signal transducer and activator of transcription (JAK/STAT) signaling pathway in leukemia, following the identification of the JAK2V617F mutation in myeloproliferative neoplasms (MPNs). The aim of this review is to summarize the latest progress in our understanding of the involvement of the JAK/STAT signaling pathway in the development of leukemia. We also attempt to provide insights into the current use of JAK/STAT inhibitors in leukemia therapy and explore pertinent clinical trials in this field.

## Introduction

### Overview of the JAK/STAT signaling pathway

**Fig. 1:**
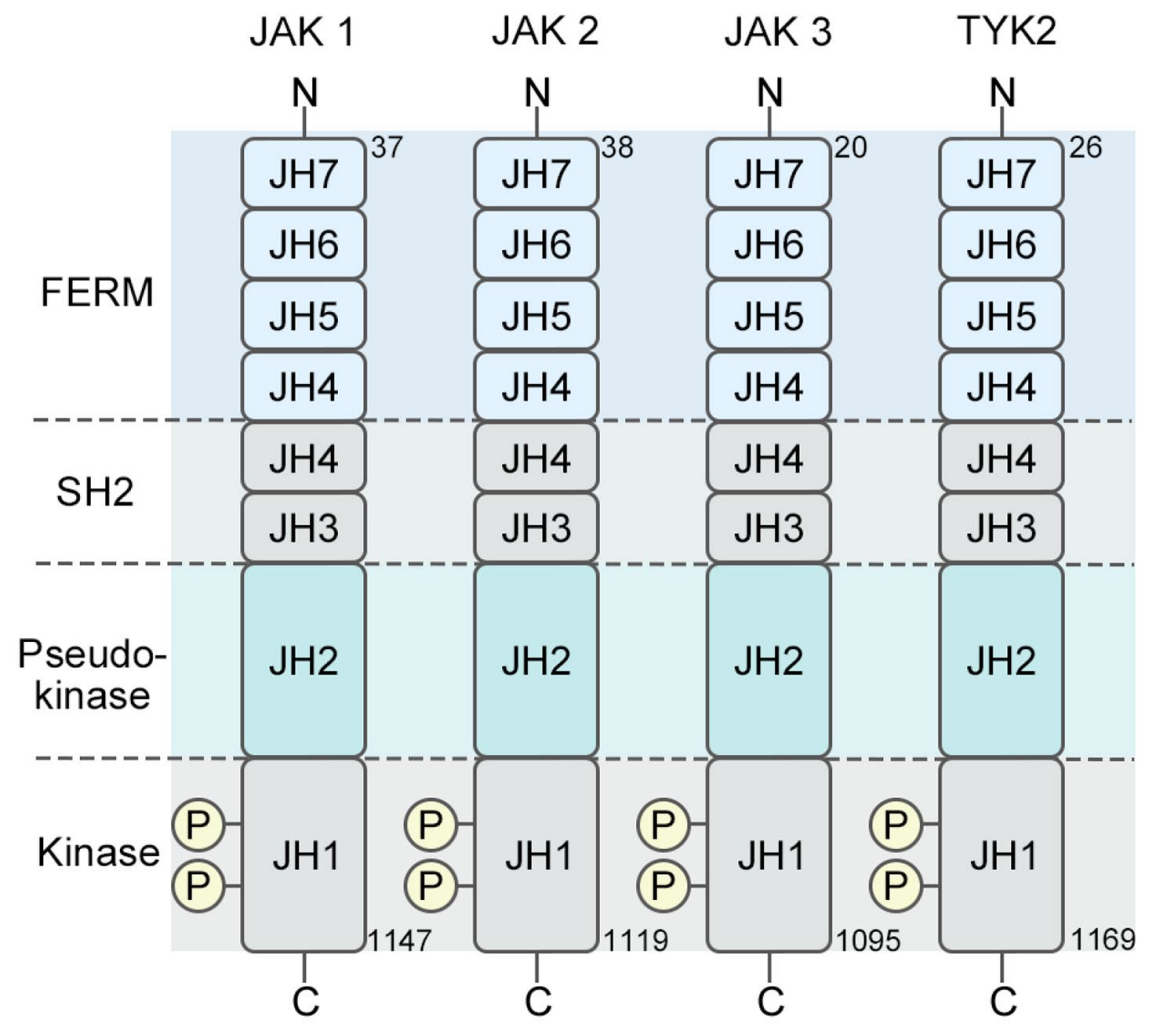
JAK structure. There are seven homologous domains in the JAK family, ranging from JH1 to JH7. The Janus homology 1 (JH1) domain functions primarily as a kinase domain. The pseudokinase domain, denoted as Janus homology 2 (JH2), exhibits kinase-like features but lacks typical kinase activity. The SH2 domain is formed by a combination of JH3 and JH4 domains, facilitating protein-protein interactions. Consisting of JH5, JH6, and a segment of the JH4 domains, the protein 4.1, ezrin, radixin, moesin (FERM) domain is involved in membrane attachment, protein interactions, and the modulation of JAK activity [6–8]

**Fig. 2:**
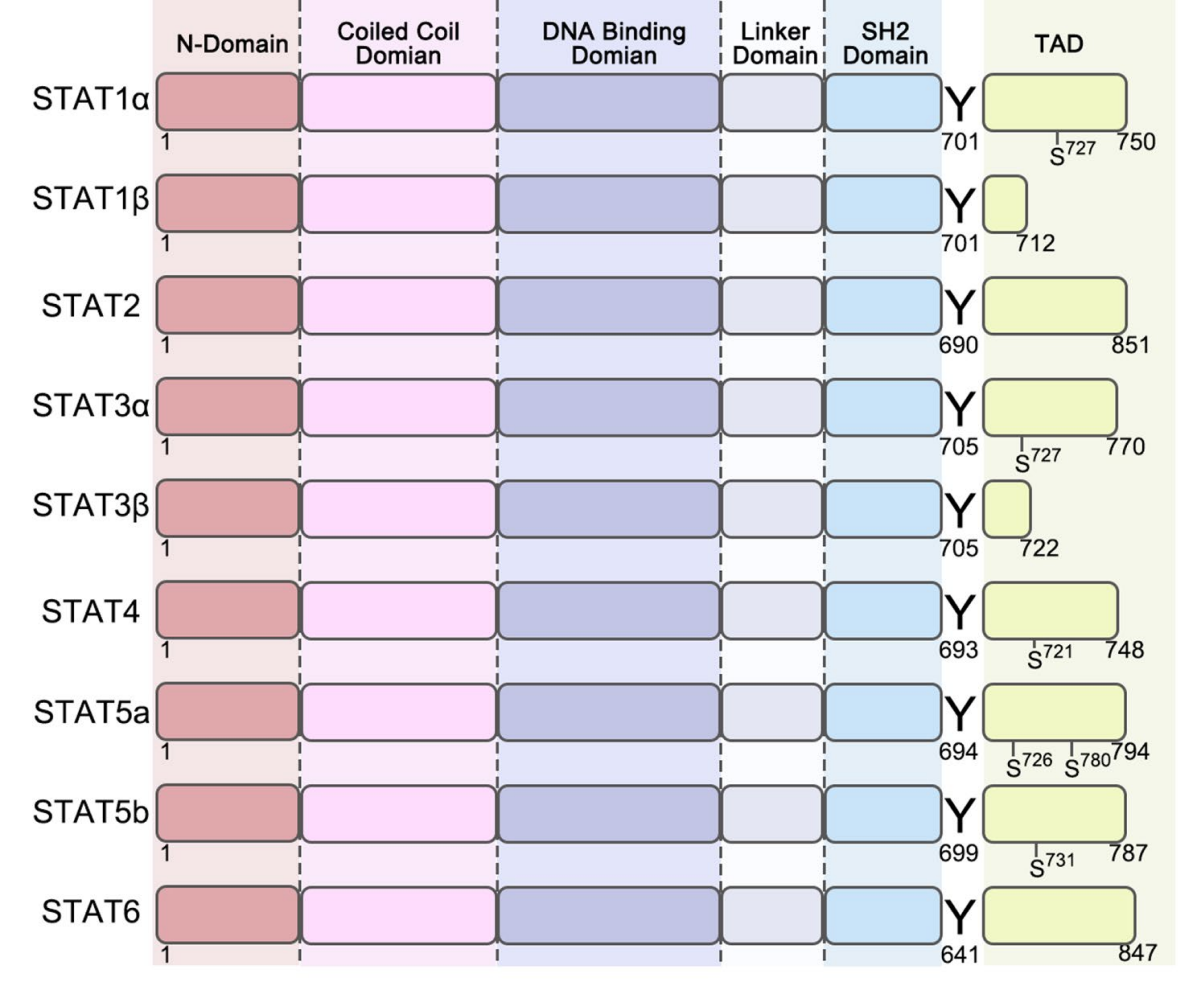
STAT structure. The STAT protein family is composed of six distinct members: STAT1, which possesses two splicing variants (STAT1α and STAT1β), STAT2 and STAT3, which also include two splicing variants (STAT3α and STAT3β), and STAT4, STAT5a, STAT5b, and STAT6. The length of STAT proteins is approximately between 750 and 900 amino acids. Their structural components consist of the following domains: N-terminal domain, coiled-coil domain, DNA-binding domain, junction domain, SH2 domain, and transcription activation domain (TAD), starting with the N-terminus and extending to the C-terminus [9–11]

The JAK/STAT signaling pathway was discovered in 1989 [1]. It is an intracellular signal transduction pathway that is widely expressed and plays an essential role in many critical biological processes, including immune system control, cell division, differentiation, and apoptosis [2]. This pathway encompasses receptor-ligand complexes, Janus kinases (JAKs), signal transducer and activator of transcription (STAT) proteins, and suppressors of cytokine signaling/cytokine-inducible Src homology 2 (SH2)-containing (SOCS/CIS) protein family, which play a critical role in finely modulating the function of the JAK/STAT pathway [3]. The JAK family includes JAK1, JAK2, JAK3, and TYK2 (Fig. 1), and the STAT family includes STAT1, STAT2, STAT3, STAT4, STAT5a, STAT5b, and STAT6 [4] (Fig. 2). The SOCS/CIS family of proteins includes SOCS1–SOCS7 and CIS [5]. Detailed categorization reveals the interplay of elements within the JAK/STAT pathway, highlighting its significance in maintaining cellular balance and coordinating cellular functions.

### Activation and inhibition of the JAK/STAT signaling pathway

**Fig .3:**
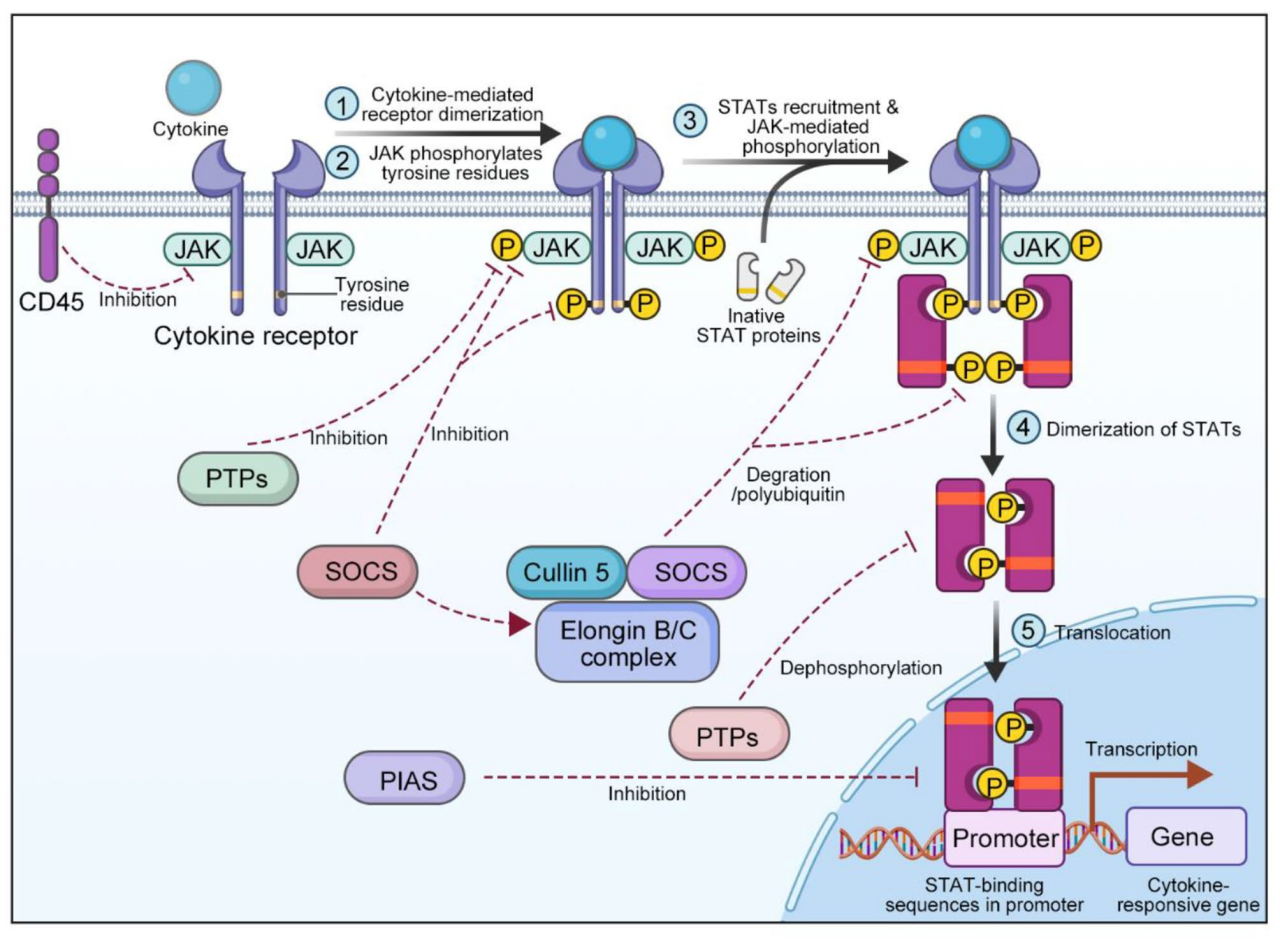
Activation and inhibition of the JAK/STAT signaling pathway. Black arrows denote the activation process, and red dashed arrows indicate the inhibition process

In the initial phase, various ligands, including interferons and interleukins (IL), bind to cell surface receptors, triggering receptor dimerization. This dimerization brings JAKs into close proximity to the receptors. Next, JAKs mutually phosphorylate each other at tyrosine residues. When JAKs are phosphorylated, phosphate groups are added to tyrosine residues on the receptor, which allows STATs to bind to the phospho-sites of the receptors with their SH2 domains. This connection causes STATs to bind to the receptor’s tyrosine-phosphorylated domains, resulting in JAK-induced tyrosine phosphorylation of STATs. The phosphorylation ultimately results in the separation of STATs from the receptors [12]. The phosphorylated STATs then translocate from the cytosol to the nucleus. This translocation is facilitated by interactions with importins, which are specific proteins that exhibit affinity for distinct STATs. Finally, the nuclear translocation of STATs triggers the transcriptional activation of specific genes of interest [13]. Inhibition of the JAK/STAT signaling pathway is governed by three principal categories of factors: SOCS/CIS, protein inhibitors of activated STATs (PIASs), and protein tyrosine phosphatases (PTPs) [14, 15]. The SOCS/CIS protein family consists of crucial molecules, including CIS, SOCS1, SOCS2, SOCS3, SOCS4, SOCS5, SOCS6, and SOCS7, that oversee the activity of the JAK/STAT signaling pathway [16, 17]. Once in the nucleus, STATs activate the SOCSs, which in turn negatively regulate the binding between STATs and receptors, establishing a feedback inhibition loop [18]. Specifically, the SOCS/CIS family enforces negative control over the JAK/STAT pathway via three principal mechanisms: (1) affinity for tyrosine kinase receptors, impeding STAT recruitment [19]; (2) direct interaction with JAK to impede its kinase activity [20]; and (3) formation of a complex involving elongin B/C and cullin5, which leads to the breakdown of JAK or STAT proteins when they are tethered to the SOCS protein via polyubiquitination, subsequently resulting in their proteasome-mediated degradation [21]. Moreover, PIASs that prevent STAT dimerization include PIAS1, PIAS3, PIASx, and PIASy [22]. PTPs also exert inhibitory effects by interfering with JAK, STAT, or receptors in the JAK/STAT pathway. This interaction leads to the following consequences: (1) deactivation of the STAT dimer through dephosphorylation [23]; (2) interference with receptor-associated JAK by inducing dephosphorylation [24]; and (3) suppression of JAK phosphorylation, particularly in the context of CD45 (a PTP with a transmembrane configuration) [25] (Fig. 3).

### Roles of the JAK/STAT signaling pathway in de novo leukemia and secondary leukemia arising from myeloproliferative neoplasms (MPN)

Previous studies have suggested that mutations in the JAK/STAT signaling pathway are not solely confined to secondary leukemia originating from MPN; they are also present in de novo leukemia [26, 27]. In 1997, the initial indication of JAK2 activation in human malignancies emerged through the discovery of the Translocation ETS leukemia (TEL)-JAK2 fusion gene [28]. This fusion gene arises from a gene rearrangement involving the TEL and JAK genes, resulting in constant kinase activity observed in patients with ALL and CML [29]. In addition to this fusion gene, several other JAK2 fusion genes have been recognized, as detailed in Table 1. The role of STAT1, STAT3, and STAT5 in leukemia development was confirmed in 1995 [30]. In the STAT family, STAT5 is a key player in the regulation of normal lymphomyeloid evolution and is of importance in leukemia pathogenesis by interacting with the adenosine triphosphate-binding site [31]. Additionally, STAT1 functions as an oncogenic promoter in leukemia progression [32]. Within the domain of hematopoietic malignancies, the improper activation of STAT3 and STAT5 has been intricately linked to gene regulation and the processes of chromatin remodeling [33]. Moreover, there is evidence suggesting that the activation of STAT5B plays a pivotal role in leukemia induced by the breakpoint cluster region–Abelson oncogene (BCR/ABL) [34]. These findings highlight the intricate role of STAT5 in various forms of leukemia and emphasize the clinical implications of STAT5 mutations. MPN include primary myelofibrosis (PMF), essential thrombocythemia (ET), and polycythemia vera (PV). The transformation of MPN to leukemia is a notable concern in hematology. This phenomenon, referred to as leukemic transformation or leukemic evolution, involves the transformation of MPN, marked by irregular and excessive multiplication of blood cells, to acute leukemia, which is distinguished by a fast buildup of immature and dysfunctional blood cells [35]. Since its identification in 1989, the JAK/STAT signaling pathway has attracted substantial interest by researchers [36]. Moreover, research has shown the critical involvement of the JAK/STAT signaling pathway in hematopoiesis, and its association with numerous malignancies is well established [37]. A significant advancement took place when researchers identified the frequent p.V617F mutation within the pseudokinase (JH2) domain of JAK2, particularly in MPN [26]. Over the course of time, numerous studies have recognized the JAK2 V617F mutation as a main driver in the development of PV. Several investigations have disclosed a connection between the phases of PV and the degree of JAK2 V617F mutation expression at the hematopoietic stem cell (HSC) and its progenitor [38–40]. Moreover, the JAK2 V617F mutation was detected in over 50% of individuals diagnosed with essential thrombocythemia (ET). This genetic alteration was also associated with adverse clinical and laboratory features among this patient cohort [41]. Additionally, the JAK2 V617F mutation was identified in nearly 50% of patients with PMF, and its presence was linked to both disease advancement and histological characteristics in this patient population [42]. Several studies have also identified that elevated JAK2V617F expression contributes to the progression of disease to leukemia in MPN [43–46]. Apart from the JAK2 V617F mutation, additional genetic alterations have been recognized as linking to the transition to leukemia or treatment inefficacy. Specifically, TP53 mutation was found to have an impact on leukemia transformation from MPN [47, 48]. DNA methylation also contributed to leukemia transformation from MPN [49]. Mutations in transcription factors also played a role in leukemia transformation from MPN [50]. These studies suggest a rationale for exploring the synergistic effect of JAK/STAT mutations and other molecular factors in fostering the development of secondary leukemia originating from MPN.

**Table 1:** JAK2 fusion gene in leukemia

Disease Category	Fusion	Specific Disease	Reference
Myeloproliferative disorder	TEL-JAK2	aCML	[51]
	PCM1-JAK2	aCML	[52]
	PCM1-JAK2	aCML	[53]
	BCR-JAK2	aCML	[54]
Acute Leukemia	PCM1-JAK2	AML	[55]
	TEL-JAK2	ALL	[56]
	PCM1-JAK2	TPALL	[57]
	ETV6-JAK2	ALL	[29]
Chronic Leukemia	ETV6-JAK2	CML	[58]

aCML, atypical chronic myeloid leukemia; TPALL, T-cell precursor ALL PCM1-JAK2: Pericentriolar Material 1-Janus Kinase 2 ETV6-JAK2: also known as TEL-JAK2

**Table 2:** JAK/STAT mutations in leukemia

JAK/STAT mutations and resulting disorders			
JAK/STAT	Mutation	Disease	Reference
JAK1	S703I	ALL	[59]
	T478S	AML	[60]
	V623A	AML	[60]
JAK2	JAK2/REED	AML	[61]
	V617F	AML	[62]
	K607N	AML	[63]
	T875N	AML	[64]
JAK3	A572VV722IP132T	AMKL	[62]
	M511I	PLL	[65]
	V722I	AMKL	[66]
	A572V	AMKL	[66]
	P132T	AMKL	[66]
STAT3	Y640F	LGLL	[67]
	D661Y	LGLL	[67]
	D661V	LGLL	[67]
	N647I	LGLL	[67]
		ATL	[68]
		T-LGLL	[69]
		AML	[70, 71]
STAT5		CML	[72, 73]
		AML	[74, 75]
		Ph + ALL	[76]
		B-ALL	[77]
STAT5a		TCL	[78]
STAT5b		T-PLL	[79]
	Y665F	LGLL	[80]
	N642H	LGLL、TCL	[81]
	N642H	T-ALL	[82]

AMKL, acute megakaryoblastic leukemia; PLL, prolymphocytic leukemia; LGLL, large granular lymphocytic leukemia; ATL, Adult T cell leukemia TCL, T cell leukemia T-LGLL, T cell large granular lymphoblastic leukemia T-ALL, T-cell acute lymphoblastic leukemia B-ALL, B-cell acute lymphoblastic leukemia T-PLL, T cell prolymphocytic leukemia Ph + ALL, Philadelphia chromosome-positive ALL.

## Crosstalk between JAK/STAT signaling pathway and p53 pathway in leukemia

**Fig. 4:**
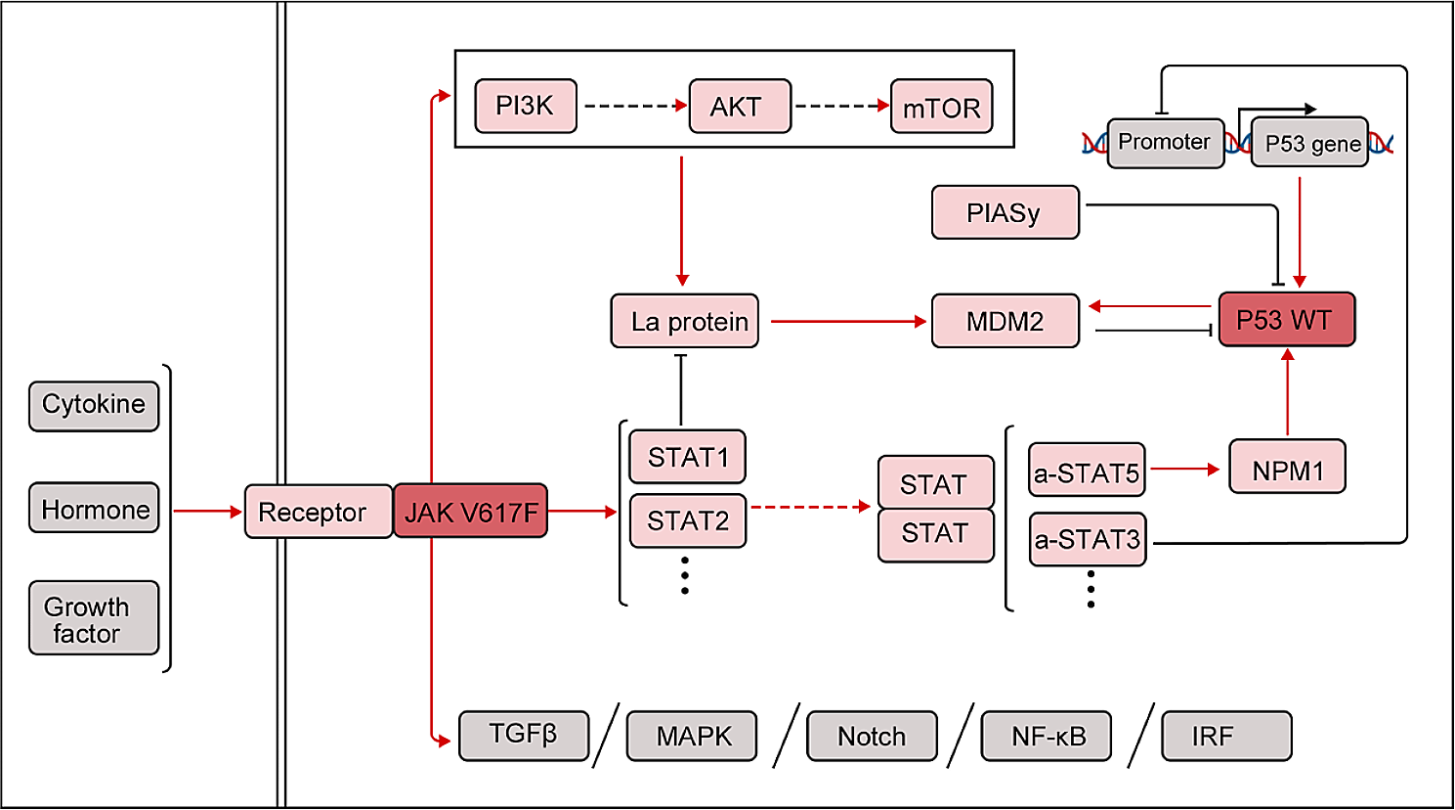
Interaction between STATs and p53. Black line denotes inhibitory regulation, and red line indicates stimulatory regulation

Multiple studies have provided compelling evidence of reciprocal signaling crosstalk between the STAT pathway and the regulatory activities of p53 [83–85] (Fig. 4). In hematological malignancies, the frequent mutation of JAK2V617F protein activates the PI3K/Akt/mTOR pathway, which in turn results in the upregulation of La protein and, subsequently, an elevated level of murine double minute 2 (MDM2). This upregulation facilitates p53 degradation [86]. Moreover, STAT1 exerts a suppressive effect on MDM2, thereby stabilizing p53 [87]. By contrast, the active STAT3 gene engages with the promoter region of TP53, resulting in the inhibition of p53 expression and potentially instigating oncogenic transformation even in the absence of TP53 mutations [88]. Additionally, p53 and persistent STAT3 activation were found to be engaged in mutual negative regulation [89]. Meanwhile, wild-type p53 represses STAT5 signaling triggered by cytokines, whereas persistent STAT5 activation disrupts the stabilizing influence of nucleophosmin on p53, ultimately resulting in the loss of p53 regulation [84]. Moreover, Protein inhibitor of activated STAT Y (PIASy) exerts a negative regulatory effect on p53 [90]. Given the frequent inactivation of p53 in AML, there is compelling evidence for an association between STAT factors and AML development.

### Rationale for developing JAK/STAT inhibitors

**Fig. 5:**
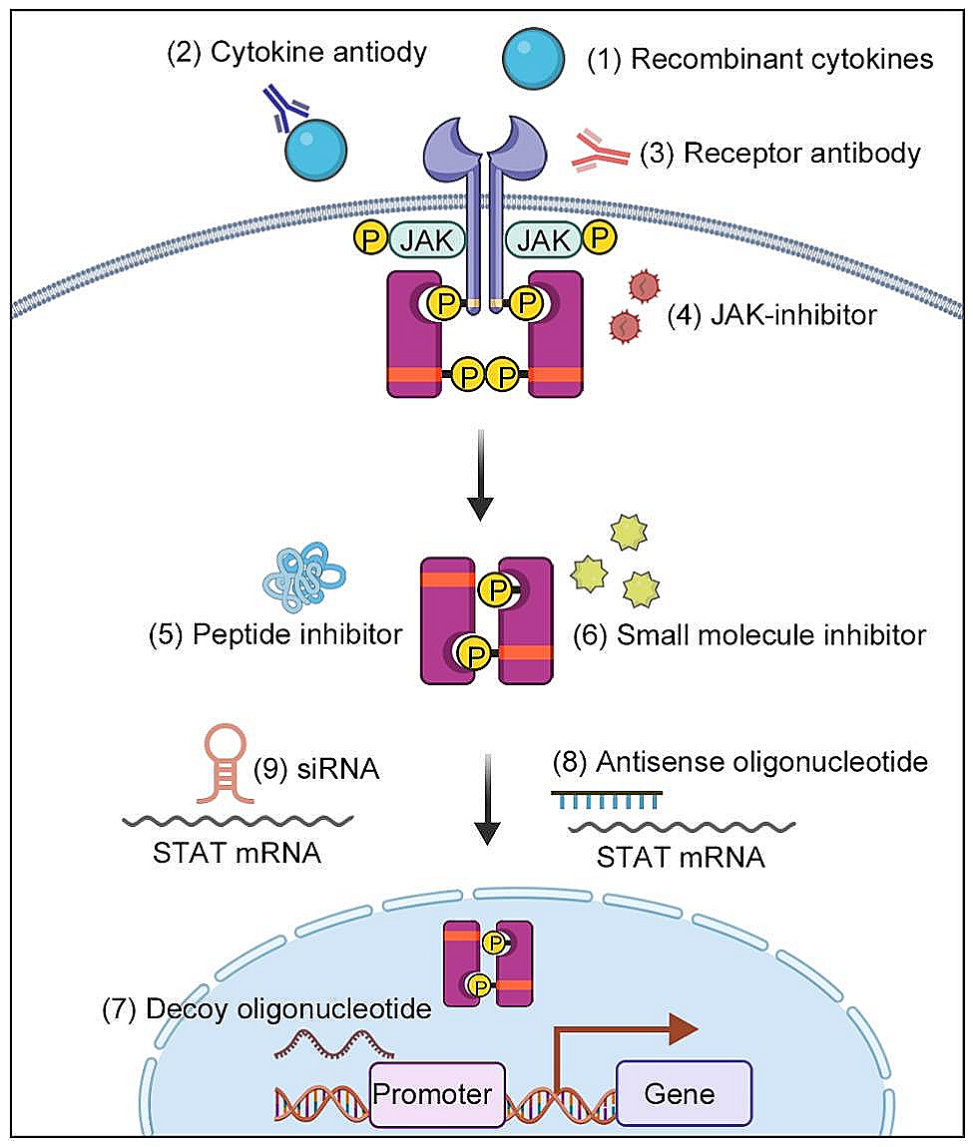
Methods for inhibiting the JAK/STAT signaling pathway. These methods include (1) recombinant cytokines, (2) cytokine antibodies, (3) receptor antibodies, (4) JAK inhibitors targeting JAKs, (5) peptide inhibitors, (6) small-molecule inhibitors, (7) decoy oligonucleotides, (8) antisense oligonucleotides, and (9) siRNAs targeting STATs

In recent decades, the rapid advancement of molecular biology has led to a growing recognition of the significance of targeting genes and proteins implicated in the pathogenesis of leukemia. Notably, the approval of imatinib by the FDA in 2001, targeting BCR-ABL, marked a pivotal development, followed by the recognition of several other targeted drugs such as Ivosidenib and Enasidenib, targeting the Isocitrate dehydrogenase gene (IDH), among others. In comparison to traditional chemotherapy, targeted treatments exhibit heightened specificity and efficacy. Furthermore, the tolerability of adverse effects associated with targeted treatments surpasses that of traditional chemotherapy. Additionally, the oral administration of most targeted drugs, as opposed to intravenous administration, contributes to increased patient adherence to medical advice. Considering the prevalence of mutations within the JAK/STAT signaling pathway in leukemia, there arises a compelling need to target this pathway for effective leukemia treatment. The pertinent mutations within the JAK/STAT pathway are succinctly presented in Table 2. Concurrently, we have compiled various therapeutic approaches aimed at targeting the JAK/STAT signaling pathway to provide a comprehensive overview of its targeting (Fig. 5). These methods contribute to the therapeutic control of the JAK/STAT signaling pathway in different diseases.

### JAK/STAT inhibitors in leukemia

JAK inhibitors effectively inhibit the enzymatic activity of JAKs by binding to their active sites. This inhibition disrupts cytokine signal transduction, preventing the downstream effects of cytokines and ultimately altering cellular responses. Promising research has demonstrated the potential of JAK inhibitors for the treatment of leukemia. For example, a 2013 study showed that the inhibition of JAK2 using TG101209 and the inhibition of both JAK1 and JAK2 with INCB18424 in leukemic mice substantially decrease tumor size and improve survival outcomes, which are antecedents of the acquisition of genetic alterations [91]. Furthermore, a phase II clinical trial involving patients diagnosed with chronic neutrophilic leukemia and atypical myeloid leukemia has revealed that ruxolitinib is well tolerated and yields a favorable response rate [92]. These findings imply that JAK inhibitors hold promise as potential therapies for leukemia and related conditions. Ongoing research is expected to delve further into their efficacy and safety in clinical settings. By contrast, STAT inhibitors interact with STAT proteins, thereby inhibiting their ability to regulate gene transcription in cells. This inhibition disrupts cellular processes that depend on STAT-mediated gene expression. A recent study published in the Journal of Leukemia showed a combination strategy involving JAK/STAT inhibitors, an inhibitor directly targeting the anti-apoptotic B-cell lymphoma 2 (BCL2) protein, and Lysine-specific demethylase 1 (LSD1) inhibitors, which demonstrate synergistic effects in selectively targeting ETP-ALL in a mouse model [93]. Inhibition of STAT3 has also been explored as a therapeutic strategy. Specifically, STAT3 inhibition, accomplished by using a STAT3-derived phosphopeptide known as Pro-pTyr-Leu-Kys-Thr-Kys, which competes with phosphorylated STAT3 monomers to hinder their formation into dimers, demonstrates effectiveness in triggering apoptosis [94]. In this study, it was demonstrated that inhibiting STAT3 with Pro-pTyr-Leu-Kys-Thr-Kys could effectively suppress the transcriptional activity of STAT3. Nevertheless, this agent requires further scrutiny as its metabolic susceptibility and cellular permeability are still undergoing clinical testing. Likewise, in another study, the inhibition of STAT5 demonstrated potent anti-leukemic activity [95]. Currently, the Food and Drug Administration (FDA) has officially approved JAK/STAT inhibitors for the management of various pathological conditions, including MPN, graft-versus-host disease, pancreatic cancer, rheumatoid arthritis, and coronavirus disease 2019 [96]. Several JAK inhibitors, including abrocitinib, ruxolitinib, fedratinib, pacritinib, tofacitinib, baricitinib, and upadacitinib, have received FDA approval. Tofacitinib targets both JAK1 and JAK3; baricitinib and ruxolitinib target both JAK1 and JAK2; and upadacitinib and abrocitinib specifically target JAK1. Fedratinib and pacritinib are selective agents for JAK2. Furthermore, ex vivo experiments carried out on AML cell lines have shown that impeding JAK2-mediated phosphorylation of STAT5 and STAT3 strongly reduces cell development and induces apoptosis [97]. Although some JAK inhibitors have obtained FDA approval, none of them are presently sanctioned to treat leukemia. We have compiled a summary of the clinical trials involving JAK inhibitors used in leukemia from https://clinicaltrials.gov in Table 3. Simultaneously, researchers have focused on creating inhibitors with a direct impact on STATs. Among the STAT family members, STAT5 and STAT3 have received considerable attention in ongoing research. STAT inhibitors are primarily categorized into three main classes: peptides, peptidomimetics, and small-molecule inhibitors constituting the majority. In addition, multiple STAT inhibitors that target various components, such as the SH2 domain, mRNA, or DNA-binding domain (DBD), in patients with AML have been tested in clinical trials [98]. These inhibitors encompass Niclosamide, fludarabine, C188-9, SD-36, AZD9150, CpG-STAT3-siRNA, CpG-STAT3dODN, AC-4-130, BP-1-107, BP-1-108, OPB-51,602, OPB-111,077, and BBI-608. Notablely, Niclosamide, OPB51602, OPB-111,077, and BBI-608, along with C188-9, SD-36, AC-4-130, BP-1-107, and BP-1-108, directly bind to the SH2 domain. On the other hand, AZD9150 and CpG-STAT3-siRNA target mRNA, while CpG-STAT3dODN targets the DNA binding domain. Of these inhibitors, common STAT inhibitors used to treat leukemia include Niclosamide, fludarabine, OPB-51,602, BBI-608, OPB-111,077, C188-9, SD-36, AZD9150, CpG-STAT3-siRNA, CpG-STAT3dODN, AC-4-130, BP-1-108, and BP-1-107. Moreover, another JAK2/STAT3 inhibitor, WP1066, currently undergoing clinical trials for the treatment of solid tumors, has demonstrated promising benefits in this context [99, 100]. Notably, some STAT inhibitors above have not been tested in clinical trials for the treatment of leukemia. We have only listed the STAT inhibitors that have been studied in clinical trials for leukemia treatment. Fludarabine is the only STAT inhibitor currently approved by the FDA for the treatment of leukemia. In this comprehensive review, we have compiled a list of STAT inhibitors sourced from https://clinicaltrials.gov for leukemia treatment, presenting the information in Table 4. The drugs featured in the table include Niclosamide selectively targeting STAT3, fludarabine inhibiting STAT1 activity, OPB-51,602 selectively targeting STAT3, OPB-111,077 specifically inhibiting STAT3, and BBI-608 inhibiting STAT3. Importantly, it is crucial to highlight that BBI-608 and fludarabine lack specificity in their inhibition mechanisms. In particular, we only listed clinical trials of fludarabine that are currently in phase 4; trials for other drugs have not advanced to phase 4. Clinical trial databases are continually updated, and new trials are regularly initiated. Therefore, for a comprehensive and up-to-date understanding of ongoing research in this field, regular consultation of clinical trial databases and relevant scientific literature is advised.

**Table 3:** Utilization of JAK inhibitors in clinical trials

Leukemia Subtype	Clinical trial	Phase	JAK Inhibitor
AML	NCT04282187	2	Ruxolitinib
AML	NCT02532010	2	Pacritinib
AML	NCT02323607	1	Pacritinib
AML	NCT03878199	2	Ruxolitinib
AML	NCT04282187	2	Pacritinib
AML	NCT01620216	2	Pacritinib
AML	NCT03874052	1	Ruxolitinib
ALL	NCT02723994	2	Ruxolitinib
CLL	NCT03041636	2	Ruxolitinib
CLL	NCT02677948	2	Pacritinib
CLL	NCT03601819	1	Pacritinib
CML	NCT01751425	1	Ruxolitinib
CML	NCT03654768	2	Ruxolitinib
CML	NCT05127174	2	Fedratinib
CML	NCT04955938	1	Fedratinib
CML	NCT02564536	1	Pacritinib
CML	NCT02469415	2	Pacritinib
TCLGLL	NCT05592015	2	Ruxolitinib
ATCL	NCT01712659	2	Ruxolitinib
TKI Resistant Philadelphia Leukemia	NCT01914484	2	​Ruxolitinb
AML ALL CLL	NCT00674479	2	Ruxolitinib

T-LGL, T-cell large granular lymphocytic leukemia; ATL, adult T cell Leukemia, TKI, tyrosine kinase inhibitor

**Table 4:** Utilization of STAT inhibitors in clinical trials

Leukemia Subtype	Clinical Trial	Phase	STAT Inhibitor
AML ALL CLL	NCT00719836	2	OPB-51,602
AML ALL CLL	NCT01344876	1	OPB-51,602
AML CML CLL	NCT02352558	1	BBI-608
AML	NCT03063944	1	OPB-111,077
AML	NCT03197714	1	OPB-111,077
AML	NCT02926586	4	Fludarabine
CLL	NCT00220311	4	Fludarabine
CLL	NCT01271010	4	Fludarabine
CLL	NCT01271010	4	Fludarabine
ALL	NCT02024308	4	Fludarabine
AML	NCT00488709	4	Fludarabine
CLL	NCT01283386	4	Fludarabine
AML	NCT05188170	1	Niclosamide

### Exploring JAK/STAT inhibitors in clinical trials for leukemia treatment: a focus on advantages and drawbacks

In this section, our primary focus revolves around the JAK/STAT inhibitors delineated in Tables 3 and 4. The JAK/STAT inhibitors presently undergoing preclinical trials for leukemia treatment are subjected to comprehensive examination herein, encompassing an evaluation of their merits and demerits. Ruxolitinib, having obtained FDA approval in 2022 for nonsegmental vitiligo and atopic dermatitis, and in 2019 for steroid-refractory acute graft-versus-host disease (GVHD) in adult and pediatric patients aged 12 and above, is scrutinized. The initiation dosage of Ruxolitinib is contingent upon the baseline platelet count of patients. Specifically, oral administration of 20 mg twice daily is recommended if the platelet count exceeds 200 × 10^9 /L, 15 mg twice daily if the count ranges from 100 × 10^9 /L to 200 × 10^9 /L, and 5 mg twice daily if the platelet count is between 50 × 10^9 /L and less than 100 × 10^9 /L. Vigilant monitoring of platelet levels is imperative during the administration of this drug. Notably, Ruxolitinib undergoes predominant metabolism via the cytochrome P450 (CYP) enzyme CYP3A4. However, limitations manifest in its restricted compatibility with other JAK inhibitors, therapeutic biologics, or potent immunosuppressants such as azathioprine or cyclosporine. Adverse events associated with Ruxolitinib treatment encompass severe infections necessitating hospitalization (e.g., tuberculosis or fungal pneumonia), heightened rates of all-cause mortality (e.g., sudden cardiovascular death, lymphoma, and other malignancies), and thrombosis among treated patients. Pacritinib, endorsed by the FDA in 2022 for adults with high-risk or intermediate primary or secondary myelofibrosis (MF), specifically those post-essential thrombocythemia or post-polycythemia vera, and featuring a recommended dosage of 200 mg orally twice daily, is explicated. Concurrent use of strong CYP3A4 inhibitors or inducers is contraindicated. Similarly, Pacritinib’s metabolism is primarily mediated by CYP3A4. Adverse events associated with Pacritinib include peripheral edema, thrombocytopenia, diarrhea, anemia, and nausea, with breastfeeding discouraged during its use. Fedratinib, granted approval in 2019 for adult patients with intermediate-2 or high-risk primary or secondary myelofibrosis (MF) post-polycythemia vera or post-essential thrombocythemia, with a recommended daily dose of 400 mg for those with a baseline platelet count greater than or equal to 50 × 10^9 /L, is detailed. Dose adjustments are advised for patients concurrently using strong CYP3A inhibitors or with severe renal impairment. Adverse events associated with Fedratinib encompass fatal encephalopathy, gastrointestinal toxicity, anemia, hepatic toxicity, thrombosis, and secondary malignancies, with its usage prohibited in patients with thiamine deficiency. OPB-51,602, despite lacking FDA approval and being under investigation for various cancer types in clinical trials, is highlighted. A clinical trial revealed common adverse events, including anorexia, early-onset peripheral neuropathy, nausea/vomiting, fatigue, and diarrhea [101]. BBI-608, still lacking FDA approval and undergoing clinical trials for gastrointestinal cancer and other cancer types, is discussed. Adverse events linked to BBI-608 are reported to be mild, with some patients experiencing gastrointestinal adverse events [102]. OPB-111,077, showcasing potent anticancer activity by inhibiting STAT3 but lacking FDA approval, is examined based on a Phase I trial. Common adverse events observed include fatigue, vomiting, and nausea [103]. Fludarabine, FDA-approved in 2008 for treating adult patients with B cell chronic lymphoblastic leukemia, is detailed, featuring a recommended adult dose of 25 mg/m2 administered intravenously over approximately 30 min daily for five consecutive days. Adverse events associated with Fludarabine include bone marrow suppression, pulmonary toxicity, potential impairment of fertility, and fetal harm if administered to pregnant women. Its contraindication pertains to patients with severe renal failure, and a lack of specificity in its mechanism of action is noted. Niclosamide, lacking FDA approval and undergoing evaluation in clinical trials for malignancies or other diseases, has a common adverse event reported as headache [104].

### JAK/STAT inhibitors in leukemia treatment: insights from clinical trials

In this section, we examine the information of selected clinical trials investigating JAK/STAT inhibitors for leukemia treatment through a comprehensive review of PubMed. Our focus is on delineating key findings derived from these noteworthy trials. The ClinicalTrial (NCT02092324) on ruxolitinib reported that the drug is well-tolerated, showcasing an overall response rate (ORR) of 32% among patients. Remarkably, no adverse events related to ruxolitinib were observed in this trial. These promising results support the potential future use of ruxolitinib as a treatment option for patients with chronic lymphoblastic leukemia (CLL) [92]. Another noteworthy clinical trial (NCT01776723) demonstrated favorable survival outcomes and acceptable adverse events in patients with Chronic Monocytic Leukemia who received ruxolitinib. This finding further emphasizes the potential efficacy and safety of ruxolitinib in treating specific leukemia subtypes [105]. Upon conducting a search on PubMed, no relevant articles reporting outcomes from clinical trials involving the use of Pacritinib and Fedratinib in leukemia treatment were found. In the following part, we will introduce the information of some representative clinical trials in which Pacritinib and Fedratinib were applied. In NCT02677948, a multi-center study, Pacritinib was combined with Ibrutinib to treat patients with relapsed or refractory CLL. In NCT02564536, Pacritinib was combined with decitabine to treat patients with CML in a phase II study with a recommended dose of 400 mg/d. Additionally, the results from a phase III PERSIST-1 trial showed that Pacritinib was highly effective in improving constitutional symptoms among patients with MF, suggesting a promising use of Pacritinib in leukemia. Additionally, in a large RCT (NCT01437787) involving 289 adult patients (≥ 18 years of age) with intermediate-2 or high-risk myelofibrosis, splenomegaly and symptoms among patients with MF were largely improved. This may also offer a reference for the use of Fedratinib in patients with leukemia. The absence of published data on Pacritinib and Fedratinib in leukemia treatment suggests a gap in the current knowledge regarding the use of these drugs in leukemia treatment. Considering the wealth of positive clinical trial data demonstrating favorable outcomes and acceptable adverse events associated with ruxolitinib in leukemia patients, ruxolitinib might emerge as a preferable treatment option compared to other JAK inhibitors in the future. As for STAT inhibitors, a phase I study of OPB-51,602 reported that no clear treatment response was observed among patients with relapsed or refractory hematological malignancies. Unfortunately, further investigation on OPB-51,602 had to be terminated due to challenges associated with maintaining a long-term high daily dose among patients [106]. As of now, there are no articles reporting outcomes of clinical trials involving the use of BBI-608 and OPB-111,077 to treat patients with leukemia on PubMed. BBI-608 was applied in a multicenter study (NCT02352558) for patients with relapsed or refractory hematological malignancies. However, the results regarding the use of this drug in clinical trials are still limited. In a phase Ib clinical trial (NCT03197714), OPB-111,077 was applied to treat patients with AML. Likewise, relevant information on clinical trials in which this drug was applied for leukemia and other hematological disorders treatment is rare. Niclosamide, a common drug used in solid malignancies, was used to treat AML in a phase I trial (NCT05188170). Similarly, only few information regarding the use of this drug in leukemia treatment is seen. In contrast, a large randomized clinical trial (NCT 00769522) involving 564 patients demonstrated that the combination of fludarabine with cyclophosphamide and rituximab exhibited superior efficacy compared to the combination of bendamustine and rituximab [107]. Additionally, several other clinical trials support the prospective use of fludarabine as the standard treatment for patients with chronic lymphocytic leukemia (CLL) [108–110]. With long-term clinical trial data and FDA approval supporting its application in CLL patients, fludarabine might emerge as a preferable choice compared to other STAT inhibitors for leukemia treatment.

### JAK inhibitor in combination therapies for leukemia treatment

Several studies have explored the combination of JAK/STAT inhibitors with standard care regimens. Ruxolitinib, when added to an induction regimen comprising L-asparaginase, dexamethasone and vincristine in patients with Ph + acute lymphoblastic leukemia, demonstrated enhanced efficacy [111]. Another study revealed that combining ruxolitinib with Bcl-2/Mcl-1 inhibitors exhibited a synergistic killing effect on leukemia cells [112]. Furthermore, the synergistic inhibition of leukemia burden by combining a Bcl-2 inhibitor and ruxolitinib was demonstrated in a murine model in separate research [113]. In a comprehensive study, 14 dysregulated network nodes involving apoptosis pathways, Ras/MAPK, JAK/STAT and other significant processes were identified. The co-targeting of these regulated network nodes presented an improved antileukemia effect [114]. This discovery also provides a rationale for combining JAK/STAT inhibitors with other drugs. Additionally, the combination of JAK inhibitors with standard care drugs in leukemia was tested in clinical trials such as NCT03874052, NCT02973711, and others. Indeed, these studies provide hope for the combination of ruxolitinib with other drugs for leukemia patients as standard therapy in the future. Nevertheless, we did not uncover articles on PubMed reporting the clinical outcomes of the combination of Fedratinib or Pacritinib with other drugs for the treatment of patients with leukemia. Further efforts and research are still needed to validate and optimize these treatment strategies.

## Conclusion and future considerations

During the last 25 years, after the identification of the JAK2V617F mutation in MPN, multiple mutations within the JAK/STAT signaling pathway have been known to contribute to the onset of leukemia. Consequently, a number of JAK/STAT inhibitors have been created to treat patients harboring these mutations. In this review, we delved into the intricate biological involvement of the JAK/STAT pathway in leukemia and introduced current JAK/STAT inhibitors, along with their respective clinical trial contexts. However, several questions require further investigation. First, the specific regulatory mechanisms controlling the JAK/STAT signaling pathway stay unknown because of their intrinsic complexity. Second, the mechanisms by which JAK/STAT inhibitors exert their inhibitory effects on leukemia require further investigation. Third, the relationship between the JAK/STAT signaling pathway and the various subtypes of leukemia must be thoroughly investigated. Fourth, JAK/STAT signaling pathway interactions with other signaling cascades need further clarification. Fifth, the current FDA-approved JAK/STAT inhibitors for leukemia treatment are limited. Ongoing research is necessary to fill this gap in knowledge. And sixth, while the enhanced killing effect on leukemia cells has been identified when combining JAK inhibitors with other drugs, there is still a considerable distance to cover before this combination becomes a standard regimen for patients with leukemia. Acknowledging the current limitation on combination therapy is important. Furthermore, a broader selection of drugs requires evaluation to determine their potential impact on the JAK/STAT signaling pathway in leukemia treatment. More research is warranted on the complicated mechanisms in controlling the participation of JAK/STAT signaling pathway in leukemia evolution.

## Data Availability

Not applicable.

## Abbreviations

JH: Janus homology
SH2: Src homology
JAK: Janus kinase
FERM: protein 4.1, ezrin, radixin, moesin
STAT: signal transducer and activator of transcription
SOCS: suppressor of cytokine signaling
CIS: cytokine-inducible SH2-containing protein
PIASs: protein inhibitors of activated STATs
PTPs: protein tyrosine phosphatases
MPN: myeloproliferative neoplasms
TEL-JAK2: Translocation ETS leukemia-Janus Kinase 2
BCR/ABL: breakpoint cluster region–Abelson oncogene
AML: acute myeloid leukemia
ALL: acute lymphoid leukemia
CML: chronic myeloid leukemia
CLL: chronic lymphoid leukemia
T-ALL: T-cell acute lymphoblastic leukemia
aCML: atypical chronic myeloid leukemia
TPALL: T-cell precursor acute lymphoid leukemia
AMKL: acute megakaryoblastic leukemia
PLL: prolymphocytic leukemia
LGLL: large granular lymphocytic leukemia
T-LGL: T cell large granular lymphocytic leukemia
TCL: T cell leukemia
ATL: adult T cell leukemia
ETP-ALL: early T-cell precursor acute lymphoblastic leukemia
MDM2: murine double minute 2
PIASy: Protein inhibitor of activated STAT Y
IDH: Isocitrate dehydrogenase gene
BCL2: B-cell lymphoma 2
LSD1: Lysine-specific demethylase 1
TKI: tyrosine kinase inhibitor
FDA: Food and Drug Administration
